# Denosumab treatment for progressive skull base giant cell tumor of bone in a 14 year old female – a case report and literature review

**DOI:** 10.1186/s13052-017-0353-0

**Published:** 2017-03-29

**Authors:** Samvel Bardakhchyan, Leo Kager, Samvel Danielyan, Armen Avagyan, Nerses Karamyan, Hovhannes Vardevanyan, Sergey Mkhitaryan, Ruzanna Papyan, Davit Zohrabyan, Liana Safaryan, Lilit Sargsyan, Lilit Harutyunyan, Lusine Hakobyan, Samvel Iskanyan, Gevorg Tamamyan

**Affiliations:** 1 Department of Oncology, Yerevan State Medical University, Yerevan, Armenia; 2 Yerevan State Medical University, Muratsan Hospital Complex, Clinic of Chemotherapy, Yerevan, Armenia; 3 Armenian Pediatric Hematology and Oncology Group, Yerevan, Armenia; 4 Department of Pediatrics, St. Anna Children’s Hospital, Medical University Vienna, Vienna, Austria; 5 Children’s Cancer Research Institute (CCRI), Vienna, Austria; 6 Department of Radiotherapy, National Center of Oncology, Yerevan, Armenia; 7 Department of Radiology, Armenian-American Wellness Center, Yerevan, Armenia

**Keywords:** Giant cell tumor of bone, Denosumab, Skull base

## Abstract

**Background:**

Giant cell tumor of bone (GCT) is a rare primary bone tumor, which can metastasize and undergo malignant transformation. The standard treatment of GCT is surgery. In patients with unresectable or metastatic disease, additional therapeutic options are available. These include blocking of the receptor activator of NF-kappa B ligand (RANKL) signaling pathway, which plays a role in the pathogenesis of GCT of bone, via the anti-RANKL monoclonal antibody denosumab.

**Case Presentation:**

Herein we report on a female teenager who presented in a very poor clinical condition (cachexia, diplopia, strabismus, dysphonia with palsy of cranial nerves V, VI, VIII, IX, X, XI and XII) due to progressive disease, after incomplete resection and adjuvant radiotherapy, of a GCT which affected the cervical spine (C1 and C2) as well as the skull base; and who had an impressive clinical response to denosumab therapy. To the best of our knowledge, this is the youngest patient ever reported with a skull base tumor treated with denosumab.

**Conclusion:**

In situations when surgery can be postponed and local aggressiveness of the tumor does not urge for acute surgical intervention, upfront use of denosumab in order to reduce the tumor size might be considered. Principally, the goal of denosumab therapy is to reduce tumor size as much as possible, with the ultimate goal to make local surgery (or as in our case re-surgery) amenable. However, improvement in quality of life, as demonstrated in our patient, is also an important aspect of such targeted therapies.

**Electronic supplementary material:**

The online version of this article (doi:10.1186/s13052-017-0353-0) contains supplementary material, which is available to authorized users.

## Background

Giant cell tumor of bone (GCT) is a rare neoplasm and accounts for about 3–5% of primary bone tumors [1]. Although classified as benign, GCTs can grow locally aggressive with a high rate of local recurrence (up to 60%) when treated only by intralesional curettage. GCTs also rarely metastasize to the lungs (5%) and may undergo malignant transformation to high grade osteosarcoma in 1–3% of patients [2].

These neoplasms usually occur in young adults (aged 20–40 years) [3–5], and typically involve the epiphysiometaphyseal region of long bones (e.g., the knee region) [6]. Less than 1% of all GCTs are found in the skull (mostly arising from sphenoid or temporal bones) [7–10] and the largest series of patients with affection of the cervical spine encompassed only 22 patients [11].

The standard treatment of GCT is near complete removal of tumor with polymethylmethacrylate adjuvants avoiding mutilations [12]. GCTs at axial sites are more difficult to treat, with a higher rate of local recurrence; and skull and spine sites are sometimes deemed inoperable, because of the proximity to vital structures like major cerebral vessels [7, 11, 13]. In GCT of the skull and spine surgical debulking with adjuvant radiotherapy is a therapeutic option [14, 15].

Additional adjuvant therapeutic options are available for the use in patients with unresectable or metastatic disease; and include blocking of the receptor activator of NF-kappa B ligand (RANKL) signaling pathway, which plays a role in the pathogenesis of GCT of bone, via the anti-RANKL monoclonal antibody denosumab. GCTs histologically consist of three cell types: multinuclear osteoclast like giant cells, neoplastic stromal cells, representing the proliferative fraction and CD68 positive mononuclear histocytic cells. Neoplastic stromal cells produce receptor activator of nuclear factor kappa-B ligand (RANKL) and induce multinuclear osteoclast like giant cell precursors which express receptor activator of nuclear factor kappa-B (RANK). Denosumab blocks the RANKL-RANK interaction between stromal cells and osteoclastic giant cell precursors inhibiting their maturation and as result bone resorption. Moreover, denosumab also reduces the relative content of proliferative tumor stromal cells and also has an anti-angiogenic effect [3, 16–20].

Herein, we report on a female teenager who, after several lines of unsuccessful therapeutic approaches, had an impressive clinical response to denosumab therapy. This is the youngest patient ever reported with a skull base tumor treated with denosumab.

## Case Presentation

A 14 year-old Caucasian female complained of headaches and neck pain for 8 months. These symptoms became more severe and additionally difficulty with swallowing and discoordination occurred. Computed tomography (CT) and magnetic resonance imaging (MRI) were performed and demonstrated an irregularly shaped, inhomogeneous intra-extracranial pathologic formation in the craniovertebral region, which seemed to arise from the C2 vertebrae, mainly at the left, with anteroposterior size up to 4.4 cm, a transverse size up to 5.5 cm and a cranio-caudal size up to 5.2 cm. The tumor comprised the craniovertebral transition, decayed the bone structure of the atlanto-occipital and atlanto-axial joints (causing luxation of atlanto-occipital articulation), as well as the upper third of the processus odontoideus of C2. It penetrated into the spinal canal, narrowed the foramen magnum, compressed the medulla oblongata and the upper sections of the cervical spinal cord, and constricted the foramen of Magendie without impairment of liquorodynamics. The C2 vertebral body was fully comprised in the process and rotated around its axis. The tumor invaded into the body of the sphenoid bone and reached almost to the level of the pons. Anteriorly, it reached the nasopharyngeal region without entering the nasopharyngeal space. Immediately posterior to the tumor the arteria basilaris was passing, with no evidence of its invasion. The left vertebral artery was lost in the thickness of the tumor. Left internal carotid artery and left jugular vein abutted to the left margin of the pathologic mass. The tumor also compressed the left cerebellar hemisphere displacing it backwards and to the top. (Additional file 1: Figure S1).

Posterior craniocervical decompression was performed with occipitospondylodesis and endonasal biopsy of the C2 vertebral body. Histological and immunohistochemical examinations showed giant cell tumor of bone with local aggressive growth (CD68+, CD99+, BCL2+, Actin+, D2-40+, Ki67 5%).

Subsequently, she was referred to Moscow, where around 2.5 months later incomplete surgical resection was performed. It was reported, that after initial tracheostomy endoscopic transnasal and microsurgical transoral subtotal removal of tumor was performed. Preoperative neurological pathology included severe craniocervical pain, grade 3–4 dysphagia, hoarseness, hypesthesia of the 2nd branch of the 5th cranial nerve, and deviation of the tongue to the left. These symptoms improved partially after surgery (relieve of craniocervical pain; partial regression of symptoms of bulbar violation together with failure of 2nd branch of the 5th cranial nerve). It was reported that postsurgical CT revealed residual tumor mass at the skull base mostly at the left.

After recovering from surgery she returned back to Armenia. Since then her preoperative symptoms gradually recurred. She received 3D conformal external beam radiotherapy (EBRT) using four coplanar fields to deliver total dose of 50.4Gy in 28 fractions. During and after completion of EBRT the above-mentioned symptoms didn’t get milder, and her health condition deteriorated.

MRI performed after the EBRT (3 months after surgery) showed progressive growth of tumor reaching 5,7 × 7,1 × 6,0 cm (Additional file 1: Figure S2).

Four weeks after completion of radiotherapy, the patient presented to our clinic with severe cachexia, craniocervical pain, discoordination, swallowing problems (grade 3–4 dysphagia), taste loss, diplopia, left eye strabismus, left auricular pain with partial deafness and dysphonia. She couldn’t stick out the tongue, which was deviated to the left, had difficulties with raising shoulders and was almost unable to turn the head to the right. Neurological examination showed that cranial V, VI, VIII, IX, X, XI and XII nerves were affected.

Due to the tumor size, location and its closeness to vital structures a re-resection was considered impossible. The patient’s health condition was extremely poor and continued to worsen; therefore the treatment had to be started immediately.

The fact that several reports stated the significant efficiency of denosumab, as well as good tolerability of this therapy prompted us to implement therapy with denosumab s/c 120 mg q4w with loading doses on days 8 and 15 of cycle 1 [3, 16, 17, 21].

Simultaneously she started taking 4000 IU oral vitamin D per day (initially her vitamin D level was 10 μg/l) and 1000 mg oral calcium.

After the first infusion of denosumab, the clinical condition improved significantly, and further neurological improvements were observed with every injection of denosumab. After two injections of the drug the patient started eating and walking, taste sense partially recovered, the hearing resumed, the diplopia disappeared, and she was free of pain without pain medication. Subsequently she gained weight and after three injections of denosumab she was discharged. After four cycles of denosumab treatment a MRI was performed and confirmed tumor regression 3,2 × 6,8 × 5,2 cm (Additional file 1: Figure S3), which was suspected from the delectable clinical course. Unfortunately, CT imaging before and after denosumab therapy is not available; and bone formation around the tumor cannot be assessed.

The patient is tolerating the denosumab treatment very well without any severe adverse effects (lowest Ca^2+^ level was 0.91 mmol/l; normal range: 1.12 to 1.32 mmol/l). She performs daily activities without any difficulties and returned to school. At last presentation the swallowing problems are almost eliminated (dysphagia grade 1), strabismus disappeared, her voice has partially recovered, and she has no difficulties with shrugging. However, some problems with turning the head to right remain. The tongue can be sticked out only slightly, and it is still deviated to the left.

## Discussion

Giant cell tumor of bone most often occurs in young adults and involves the epiphysiometaphyseal region of long bones [3–6]. Only a minority of patients present with tumors in axial sites [7–11], and such manifestations are exceptionally rare in pediatric patients. To the best of our knowledge only 16 (including our case) pediatric patients (aged between 7 weeks and 17 years) with GCTs of the cervical spine (Table 1) and 20 children and adolescents with GCTs of the skull base (Table 2) have been reported [11, 22–35]. Most of them were treated with surgery and/or radiotherapy. One patient was reported to have been treated with chemotherapy (Table 1). Many of these patients were treated in the pre-RANKL inhibitors era (i.e., in the last century).

**Table 1:** Cases of cervical spine GCTs in pediatric patients reported in the English literature

Article	Patient, Age, Sex	Location	Therapy	Tumor recurrence	Last status	Follow-up period (in years)
Dahlin DC, Cancer. 1977;39(3):1350–1356 [27]	3 patients, one 14 and two 17 years, all female	1.C2 2.C2 3. C4	All subtotal resection, one patient additional radiotherapy	No	ANED	1. 1,4 years 2. 4,8 years 3. 8,7 years
Di Lorenzo N, Neurosurgery. 1989;24(1): 37–42 [28]	1 patient, 17 years, Sex - N/A	C2	Total resection	No	ANED	4 years
Hart et al., Spine (Phila Pa 1976). 1997;22(15):1773–1782 [29]	1 patient, 14 year, female	C2	Intralesional curettage twice	Multiple recurrences	AWD	19 years
Honma et al., Spine (Phila Pa 1976). 1989;14(11):1204–1210 [30]	1 patient, 17 years, female	C1-C2	Subtotal resection, radiotherapy (50Gy), chemotherapy	Recurrence both after surgery and after radiotherapy	DWD	2 years
Junming et al., Spine (Phila Pa 1976). 2008;33(3):280–288 [11]	1 patient, 17 years, female	C7	En-bloc resection	No	ANED	7,7 years
Mirra et al., Clin Orthop Relat Res. (154):228–233 [31]	1 patient, 17 years, female	C2	Cryosurgery and radiotherapy (46Gy)	No	ANED	2,5 years
Sanjay et al., Bone Joint J. 1993;75-B(1) [32]	4 patients, 14, 15 and two 17 years, all female	1. C2 2. C7 3. C2 4. C4	1. Anterior and posterior excision, additional surgery for recurrences 2. Anterior excision 3. Posterior excision, additional surgery for recurrence and radiotherapy (45Gy) 4. Posterior excision	1. Multiple recurrences 2. Multifocal lesions 3. One recurrence after first surgery 4. No	1. AWD 2. AWD 3. ANED 4. ANED	1. 4 years 2. 4 years 3. 20 years 4. 25 years
Shirzadi et al., J Neurosurg Pediatr. 2011;8(4):367–371 [33]	1 patient, 15 years, male	C2	3 resections, radiotherapy (40Gy), chemotherapy, additional surgery	No recurrence after last surgery	ANED	10 years
Teng et al., J Neurosurg. 1951;8(5):482–493 [34]	1 patient, 15 years, female	C6	Total resection and X-ray therapy	N/A	N/A	N/A
Willard et al., Ann Surg. 1938;107(2):298–302 [35]	1 patient, 9 years, female	C4	Subtotal resection	No	ANED	1 year
Our case	1 patient 14 year, female	C1-C2, clivus, sphenoid	Subtotal resection and radiotherapy	Recurrence both after surgery and after radiotherapy	AWD	1 year

**Table 2:** Cases of skull base GCTs in pediatric patients reported in the English literature

Article	Patient, Age, Sex	Location	Therapy	Tumor recurrence	Last status	Follow-up period (in years)
Bertoni et al., J Bone Joint Surg Am, 1985 Jul;67(6):890–900	1 patient, 8 years, female	Temporal	Gross total resection	Recurrence	AWD	2,5 years
Bibas-Bonet et al., Pediatr Neurol. 2003 May;28(5):392–5	1 patient, 8 years, female	Temporal, sphenoid	Radiotherapy	N/A	N/A	7 years
Carmody et al., J Comput Assist Tomogr. 1983 Apr;7(2):370–3	1 patient, 16 years, male	Sphenoid	Subtotal resection and radiotherapy	No	ANED	0,8 year
Do Amaral et al., J Craniofac Surg. 1994 Sep;5(4):254–6	1 patient, 14 years, female	Sphenoid	Gross total resection and radiotherapy	No	ANED	4 years
Elder et al., J Neurosurg. 2007 Jul;107(1 Suppl):69–74	2 patients, 2 years and 7 weeks, both female	Both temporal	Both gross total resection	No	ANED	1.1,1 years 2.0,9 years
Gupta et al., Br J Neurosurg, June 2008; 22(3): 447 –449	1 patient, 17 years, female	Occipital	Subtotal resection and radiotherapy	No	ANED	2 years
Inoue et al., World Neurosurg. 2016;91:674.e1–674.e6	1 patient, 16 years, male	Sphenoid, clivus	Subtotal resection and denosumab treatment after recurrence	Recurrence after surgery, regress with denosumab	AWD	N/A
Kamoshima et al., Neurol Med Chir (Tokyo) 2011;51(11):798–800	1 patient, 2 years, female	Frontal	Gross tumor resection	No	ANED	1,5 years
Kattner et al., Skull Base Surg. 1998;8(2):93–7.	1 patient, 9 years, female	Sphenoid	Subtotal resection and radiotherapy	No	ANED	1 year
Kishima et al., Br J Neurosurg. 2001 Apr;15(2):171–4	1 patient, 12 years, female	Sphenoid	Debulking twice and radiotherapy on recurrence(50Gy)	Recurrence after debulking, but stable after radiotherapy	AWD	5 years
Sharma et al., Cases J. 2009; 2: 74	2 patients, 17 and 12 years, male and female	1. Sphenoid 2. Temporal	1. Subtotal resection and radiotherapy 2. Gross tumor resection	No	ANED	1. 2 years 2. 1 year
Weber et al., Skull base surgery. 1997; 7(4):163–173	3 patients, 16, 11 and 10 years, male and two females	All sphenoid	All subtotal resection	Yes (in all cases)	AWD	1. 3,2 years 2. 1,5 years 3. 0,9 year
Wolfe et al., J Neurosurg. 1983;59(2):322–327	2 patients, both 16 years, female and male	1. Sphenoid, clivus, sella 2. Sella	Both subtotal resection and radiotherapy	No	ANED	1. 8 years 2. 2,5 years
Zhang et al., J Neurooncol. 2013;115(3):437–444	1 patient, 17 years, male	Sphenoid	Subtotal resection	Yes	AWD	1,6 years
Zorlu et al., J Neurooncol. 2006;76(2):149–152	1 patient, 14 years, female	Sphenoid, clivus	Debulking and radiotherapy (60Gy) on recurrence	Recurrent both after surgery and after radiotherapy	AWD	2 years

The first and the only report describing beneficial effects of denosumab in the treatment of the skull base GCT was published recently by Inoue and colleagues, describing a 16 years-old male with a relapsed GCT of the skull base treated with denosumab after failure of the surgery, resulting in marked reduction in tumor size [36].

To the best of our knowledge, our patient is the youngest patient ever reported with a large progressive GCT affecting the skull base and cervical spine, who responded well to therapy with denosumab.

As in most patients with spine and skull base GCTs, total surgical excision (which is the standard therapy for GCT) was not feasible in our patient. After subtotal excision she therefore received RT. As neurological symptoms recurred gradually after surgery and became worse during and after RT, and correlated with rapid tumor growth (documented via MRI), we considered these symptoms mainly caused by tumor growth and concluded that RT, unfortunately, was not effective. Therefore we decided to implement palliative treatment with denosumab [3, 16, 17, 21]. Initial cycles of treatment, however, showed excellent improvement in performance status; and MRI performed after 4th treatment cycles confirmed tumor regression. Radiotherapy can induce malignant transformation in GCTs. However, it is unlikely that this occurred in our patient, because the tumor has grown already before start of radiotherapy, the time-lag between radiotherapy and occurrence of malignancy is considered to take longer [15], and there was an excellent response to denosumab therapy. As complete surgical remission is crucial for long-term survival in patients with GCTs, the long-term prognosis in our patient is expected to be unfavorable. However, as she is tolerating the denosumab therapy without any severe adverse effects, we will continue denosumab therapy.

## Conclusion

Based on the experience in our patient and other similar reports [3, 16–19, 36] where denosumab was effective in reducing GCT size and brought to elimination of 90% or more giant cells we would now use denosumab upfront in order to reduce tumor size in similar situations. However, such an approach is only feasible, if surgery can be postponed and local aggressiveness of the tumor does not urge for acute surgical intervention.

Principally, the goal of denosumab therapy is to reduce tumor size as much as possible, with the ultimate goal to make local surgery (or as in our case re-surgery) amenable. However, improvement in quality of life, as demonstrated in our patient, is also an important aspect of such targeted therapies.

## Additional file

Additional file 1:
**Figure S1.** MRI at diagnosis. (A) axial, (B) coronal and (C) sagittal section of T1 weighted MRI after contrast administration revealing an intra-extracranial pathologic formation of craniovertebral region 4,4×5,5×5,2 cm in size, which was rapidly enhancing after the gadolinium injection, with decay of bones of craniovertebral region, upper third of processus odontoideus of C2 vertebrae, luxation of cervical vertebrae at the level of atlantoaxial articulation, more profoundly on the right side. Formation grows into the sphenoid bone reaching the brainstem, largely the pons, constrictes foramen of Magendie, compresses the left cerebellar hemisphere and reaches the nasopharyngeal region without entering nasopharyngeal space. **Figure S2.** MRI after radiotherapy. (A) axial, (B) coronal and (C) sagittal images of T1 weighted MRI after contrast admision performed after surgery and radiation therapy revealed recurrence of the pathologic mass of the craniovertebral junction, up to 5,7×7,1×6,0 cm in size, with central necrosiss. Signs of decay, which replaces clivus, ventral parts of C1-C2, and condyles of temporal bone. Ventrally it infiltrates retropharyngeal space, dorsally deformates and obliterates lumen of pontine cistern and cisterna magna, compressing ventral parts of brainstem. **Figure S3.** MRI after 4 cycles of denosumab. (A) axial, (B) coronal and (C) sagittal images of T1 weighted MRI after contrast administration with image subtraction (C) after 4 cycles of denosumab treatment revealing an intra-extracranial pathologic formation of craniovertebral region 3,2×6,8×5,2 cm in size, with decay of bones of atlantooccipital and atlantoaxial articulation and upper third of processus odontoideus of C2 vertebrae, with spine offset to the right. The pathological mass enhances after the gadolinium injection, with small hypointense areas, with indicate focal necrosis. The mass grows into the sphenoid bone reaching the brainstem, largely the pons, ventrally reaches nasopharyngeal region without entering nasopharyngeal space. Dorsolateral parts of formation reach condyles of temporal bone, constricts craniovertebral transition and deformates front loop of medulla. The formation is inhomogen due to small parts of cystic transformation. (DOCX 688 kb)

## Abbreviations

ANED: Alive with no evidence of disease
AWD: Alive with disease
DWD: Dead with disease
EBRT: External beam radiotherapy
GCT: Giant cell tumor of bone
MRI: Magnetic resonance imaging
N/A: Not available
OPG: Osteoprotegerin
RANKL: Receptor activator of NF-kappa B ligand
