# Supplementary material for: Denosumab treatment for progressive skull base giant cell tumor of bone in a 14 year old female – a case report and literature review
Source: Ital J Pediatr. 2017 Mar 29;43:32. doi: 10.1186/s13052-017-0353-0 (PMC5372271; doi:10.1186/s13052-017-0353-0)

**Figure S1. MRI at diagnosis.**

(A) axial, (B) coronal and (C) sagittal section of T1 weighted MRI after contrast administration revealing an intra-extracranial pathologic formation of craniovertebral region 4,4x5,5x5,2cm in size, which was rapidly enhancing after the gadolinium injection, with decay of bones of craniovertebral region, upper third of processus odontoideus of C2 vertebrae, luxation of cervical vertebrae at the level of atlantoaxial articulation, more profoundly on the right side. Formation grows into the sphenoid bone reaching the brainstem, largely the pons, constrictes foramen of Magendie, compresses the left cerebellar hemisphere and reaches the nasopharyngeal region without entering nasopharyngeal space.

**
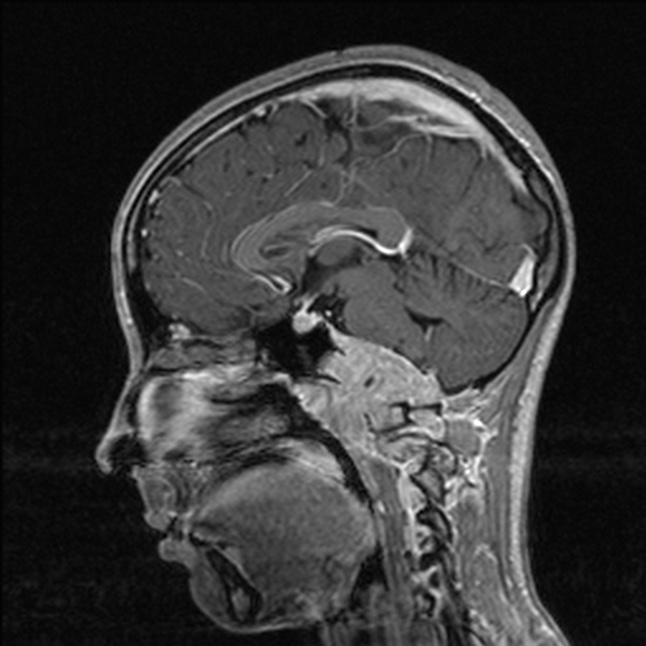
**A)

B) **
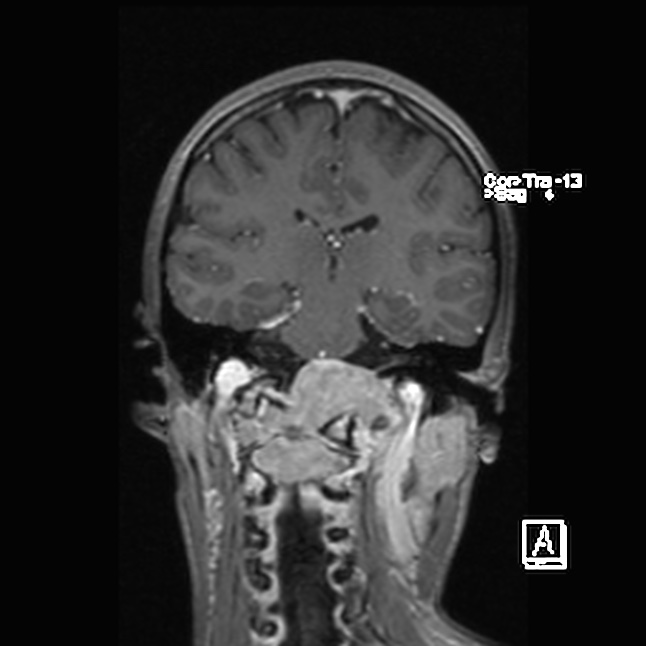
**

C) **
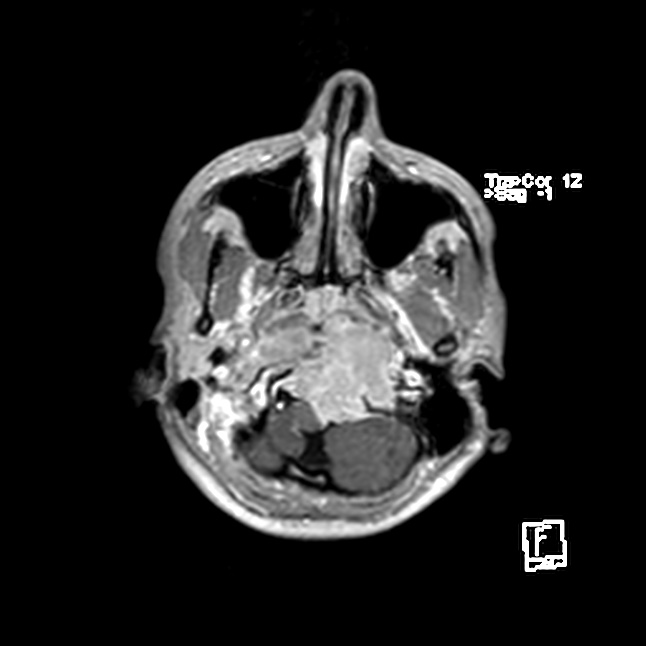
**

**Figure S2. MRI after radiotherapy.**

(A) axial, (B) coronal and (C) sagittal images of T1 weighted MRI after contrast admision performed after surgery and radiation therapy revealed recurrence of the pathologic mass of the craniovertebral junction, up to 5,7x7,1x6,0cm in size, with central necrosiss. Signs of decay, which replaces clivus, ventral parts of C1-C2, and condyles of temporal bone. Ventrally it infiltrates retropharyngeal space, dorsally deformates and obliterates lumen of pontine cistern and cisterna magna, compressing ventral parts of brainstem.

A) **
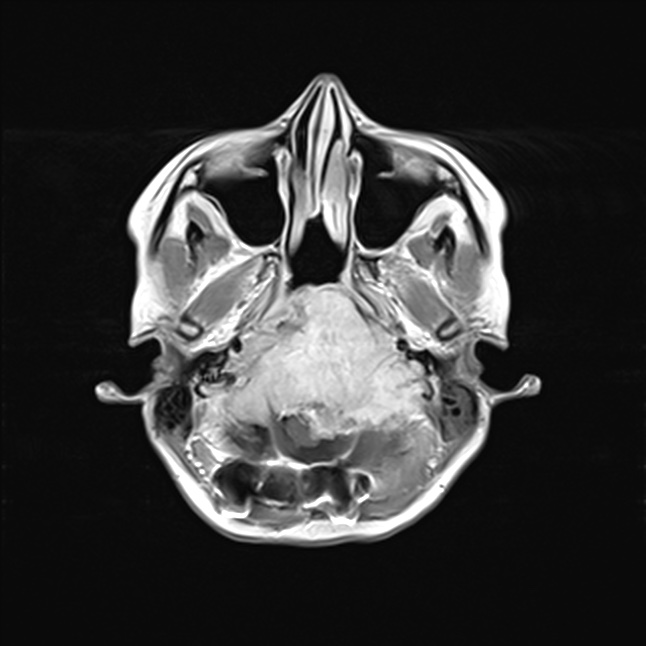
**

B)

**
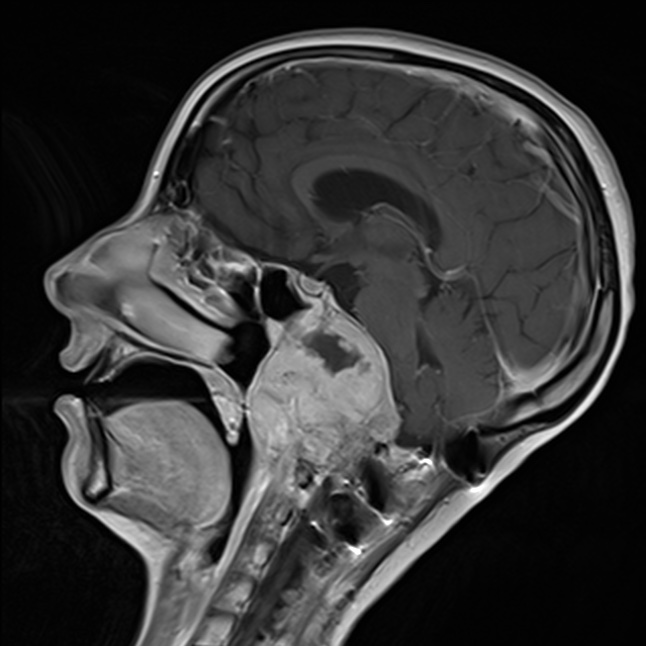
**

C)

**
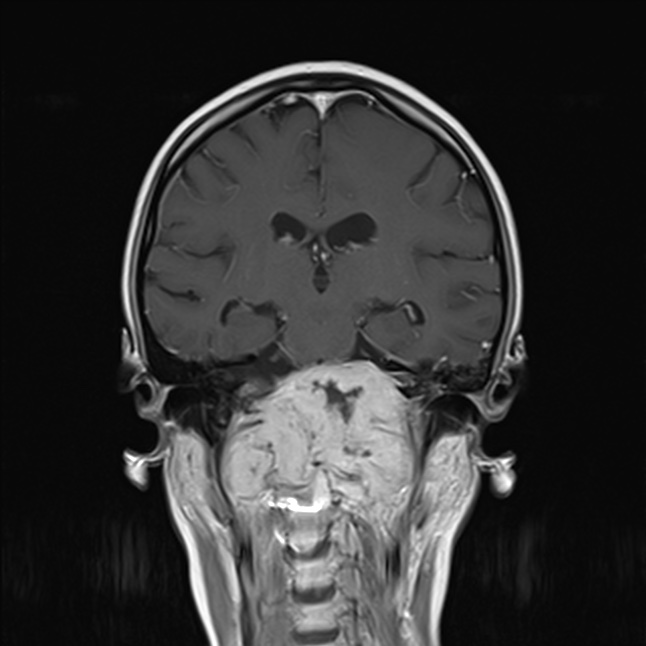
**

**Figure S3. MRI after 4 cycles of denosumab.**

(A) axial, (B) coronal and (C) sagittal images of T1 weighted MRI after contrast administration with image subtraction (C) after 4 cycles of denosumab treatment revealing an intra-extracranial pathologic formation of craniovertebral region 3,2x6,8x5,2cm in size, with decay of bones of atlantooccipital and atlantoaxial articulation and upper third of processus odontoideus of C2 vertebrae, with spine offset to the right. The pathological mass enhances after the gadolinium injection, with small hypointense areas, with indicate focal necrosis. The mass grows into the sphenoid bone reaching the brainstem, largely the pons, ventrally reaches nasopharyngeal region without entering nasopharyngeal space. Dorsolateral parts of formation reach condyles of temporal bone, constricts craniovertebral transition and deformates front loop of medulla. The formation is inhomogen due to small parts of cystic transformation.

A)


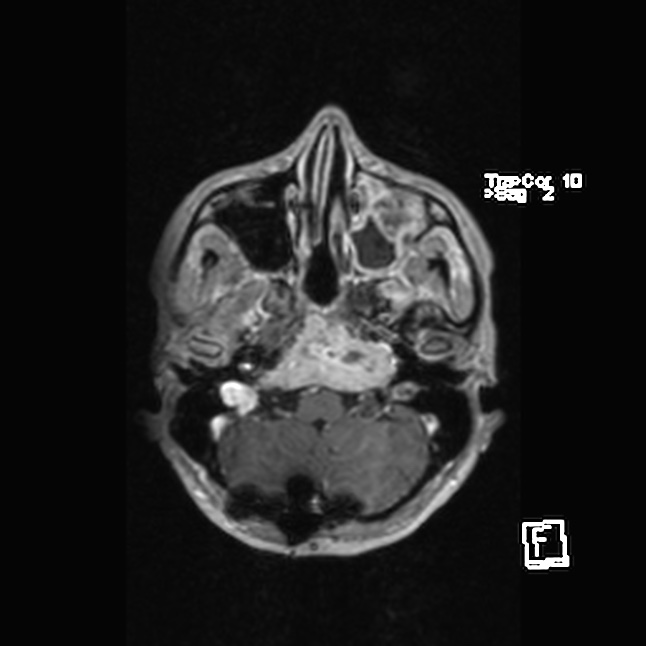


B)
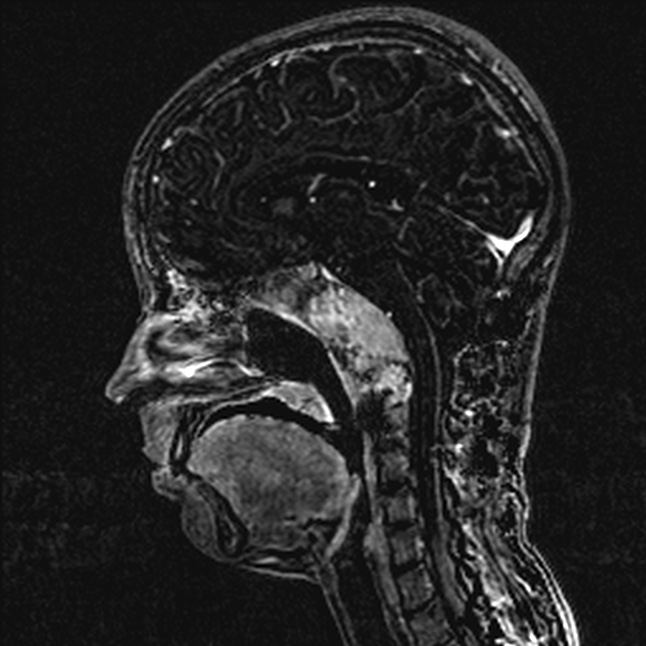


C)
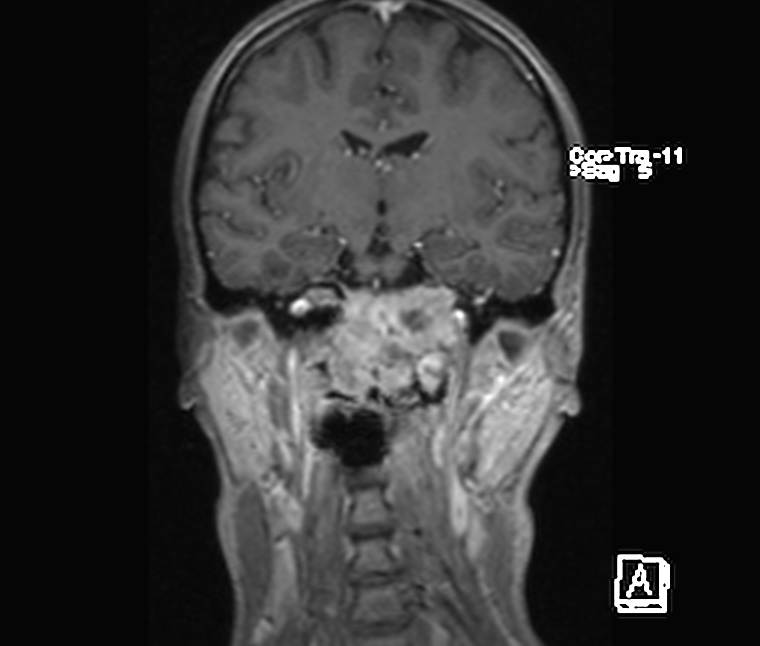

Supplement: Additional file 1: — Figure S1. MRI at diagnosis. (A) axial, (B) coronal and (C) sagittal section of T1 weighted MRI after contrast administration revealing an intra-extracranial pathologic formation of craniovertebral region 4,4×5,5×5,2 cm in size, which was rapidly enhancing after the gadolinium injection, with decay of bones of craniovertebral region, upper third of processus odontoideus of C2 vertebrae, luxation of cervical vertebrae at the level of atlantoaxial articulation, more profoundly on the right side. Formation grows into the sphenoid bone reaching the brainstem, largely the pons, constrictes foramen of Magendie, compresses the left cerebellar hemisphere and reaches the nasopharyngeal region without entering nasopharyngeal space. Figure S2. MRI after radiotherapy. (A) axial, (B) coronal and (C) sagittal images of T1 weighted MRI after contrast admision performed after surgery and radiation therapy revealed recurrence of the pathologic mass of the craniovertebral junction, up to 5,7×7,1×6,0 cm in size, with central necrosiss. Signs of decay, which replaces clivus, ventral parts of C1-C2, and condyles of temporal bone. Ventrally it infiltrates retropharyngeal space, dorsally deformates and obliterates lumen of pontine cistern and cisterna magna, compressing ventral parts of brainstem. Figure S3. MRI after 4 cycles of denosumab. (A) axial, (B) coronal and (C) sagittal images of T1 weighted MRI after contrast administration with image subtraction (C) after 4 cycles of denosumab treatment revealing an intra-extracranial pathologic formation of craniovertebral region 3,2×6,8×5,2 cm in size, with decay of bones of atlantooccipital and atlantoaxial articulation and upper third of processus odontoideus of C2 vertebrae, with spine offset to the right. The pathological mass enhances after the gadolinium injection, with small hypointense areas, with indicate focal necrosis. The mass grows into the sphenoid bone reaching the brainstem, largely the pons, ventrally reaches nasopharyngeal [file 13052_2017_353_MOESM1_ESM.docx]
